# What should be done about policy on alcohol pricing and promotions? Australian experts’ views of policy priorities: a qualitative interview study

**DOI:** 10.1186/1471-2458-13-610

**Published:** 2013-06-25

**Authors:** Andrea S Fogarty, Simon Chapman

**Affiliations:** 1 Sydney School of Public Health, The University of Sydney, Edward Ford Building (A27), Sydney, NSW 2006, Australia

**Keywords:** Alcohol advertising, Alcohol pricing, Policy, Public health, Advocacy

## Abstract

**Background:**

Alcohol policy priorities in Australia have been set by the National Preventative Health Task Force, yet significant reform has not occurred. News media coverage of these priorities has not reported public health experts as in agreement and Government has not acted upon the legislative recommendations made. We investigate policy experts’ views on alcohol policy priorities with a view to establishing levels of accord and providing suggestions for future advocates.

**Methods:**

We conducted semi-structured in depth interviews with alcohol policy experts and advocates around Australia. Open-ended questions examined participants’ thoughts on existing policy recommendations, obvious policy priorities and specifically, the future of national reforms to price and promotions policies. All transcripts were analysed for major themes and points of agreement or disagreement.

**Results:**

Twenty one alcohol policy experts agreed that pricing policies are a top national priority and most agreed that “something should be done” about alcohol advertising. Volumetric taxation and minimum pricing were regarded as the most important price policies, yet differences emerged in defining the exact form of a proposed volumetric tax. Important differences in perspective emerged regarding alcohol promotions, with lack of agreement about the preferred form regulations should take, where to start and who the policy should be directed at. Very few discussed online advertising and social networks.

**Conclusions:**

Despite existing policy collaborations, a clear ‘cut through’ message is yet to be endorsed by all alcohol control advocates. There is a need to articulate and promote in greater detail the specifics of policy reforms to minimum pricing, volumetric taxation and restrictions on alcohol advertising, particularly regarding sporting sponsorships and new media.

## Background

Alcohol consumption is recognised as a leading cause of preventable illness and a major social burden [1,2]. Risky consumption of alcohol causes hospitalisation from injury [3,4], liver, brain, and other disease [5], emergency department presentations for assault [6], and high costs for alcohol associated incidents attended by law enforcement representatives [7]. Around a third of adult Australians drink alcohol at levels that put them at risk of harm from a single drinking occasion at least once per month [8]. Of concern for the community, one in five young people report drinking at very high risk levels at least once a month, with increasing trends in hospitalisations and assault [9,10]. Taken together, alcohol consumption carries significant public and private costs [11-13].

In September 2009 the Australian government released recommendations made by the Alcohol Working Group of the National Preventative Health Taskforce, aimed at reducing consumption of alcohol in Australia and its attendant risks to health [14]. The report chronicled policy approaches consistent with international policy recommendations [15,16] and supported by a range of Australian health and medical organisations [17-21]. The recommendations focused on (i) regulating the availability of alcohol, (ii) taxation and pricing measures, (iii) drink-driving counter measures, (iv) provision of treatment services, (v) altering drinking contexts to reduce harm, (vi) regulating advertising and promotion of alcohol, and (vii) education and persuasion strategies [14]. The introduction of such preventive health policies would provide cost-effective savings to the health sector and beyond by reducing the need for treatments for alcohol related injury and disease and reducing costs associated with law enforcement [13,22,23].

Unlike policies affected by differences in state-based regulations, two of these interventions had the potential to be nationally implemented by Australia’s federal government. Namely, regulating alcohol’s price through taxation and restricting alcohol advertising and promotional activities. Pricing policies that address consumption of alcohol consist of manipulations to taxes applied to alcohol and setting minimum prices below which alcohol cannot be sold, and are supported cost-effective approaches to reducing consumption [13]. Up to date evidence and economic modelling has shown that an increase in the price of alcohol is associated with reductions in both population level consumption and overall health-care costs; effects which could be maintained by a minimum price policy in a subgroup of harmful drinkers [24]. Recently a resolution on minimum pricing was adopted by the World Medical Association adding to global agreement on policies to address alcohol’s price [25]. Another meta-analysis concluded that the effects of tax and pricing policies on reducing consumption of alcohol are larger than those achieved through other prevention policies [26]. Similarly, doubling alcohol taxes are estimated to have a considerable impact on a range of health outcomes, resulting in declines in mortality, morbidity, accidents, crime and violence [27]. Despite agreement on pricing policies among public health organisations, sections of the drinks industry in Australia have objected to the introduction of volumetric taxation and minimum pricing measures [28].

In 2008, the Australian government commissioned a review of the country’s taxation system, known as the Henry Tax Review [29]. Recommendation 71 of the review states that “all alcoholic beverages should be taxed on a volumetric basis, which, over time, should converge to a single rate, with a low-alcohol threshold introduced for all products” [30]. Beverages would thus be taxed according to the percentage of alcohol instead of the current system which applies taxes based on categories. Beer and spirits are covered by several different rates, while wine is subject to the Wine Equalisation Tax (WET), where tax is applied based on value at 29 per cent of the wholesale price, resulting in low taxes on cheap wines that can have more alcohol by volume than beer [31]. Economic modelling replacing the WET with volumetric taxation, or a tax applied at similar rates to beer, found that cheap wine would become more expensive, leading to a drop in alcohol consumption [32]. Some concerns about a volumetric tax have arisen in Australia, because consumption of beer and wine would be predicted to reduce, but consumption of spirits could increase, warranting further investigation of whether consumption patterns of particular drinks are more associated with risks to health than others [33]. Recognising this issue, some question whether a volumetric tax should be a flat, single rate as suggested by Henry, or whether there should be graded bands within the tax, with progressively higher rates of tax within each step. This, or abolition of the WET is the preferred solution for some alcohol advocates to account for the possibility that spirits might be made cheaper by a flat tax [31].

Policy proposals that would restrict or regulate alcohol advertising take the form of bans or partial bans on all alcohol advertising, restrictions on the content of alcohol advertisements, and regulating their frequency and placement. Such proposals are also considered cost-effective tools in reducing alcohol consumption [13]. Alcohol advertisements in Australia are subject to voluntary codes, including the Australian Association of National Advertisers (AANA) Advertiser Code of Ethics [34], the Commercial Television Industry Code of Practice (CTICP) [35] and an industry voluntary agreement that regulates timing and content of some advertisements, named the Alcoholic Beverage Advertising Code [36]. Evidence supporting policy proposals to regulate alcohol advertising is not as firmly established as for some other proposed measures, in part due to the improbability that controlled trials banning or reducing exposure to advertising could ever be undertaken. Nevertheless, there is good evidence for associations between alcohol advertising and increased consumption. A systematic review of longitudinal studies found that among young people, alcohol advertising is related to increased consumption and commencement of drinking in non-drinkers [37]. There is also evidence that voluntary codes of practice regarding alcohol advertisements are not effective, with various failures of self-regulation seeing ongoing exposure to advertising by minors [38-42].

In the wake of the federal government’s 2008 introduction of the ‘alcopops tax’ on ready-to-drink (RTD) pre-mixed spirits [43], their stated commitments to improving population health through the use of prevention strategies [44], a request for a discussion paper on minimum pricing from the Australian National Preventive Health Agency [45] and the Henry Review of taxation [30], the policy atmosphere seemed promising for alcohol control advocacy in Australia toward the end of the first decade of the new millennium. Collaborations of Australian health researchers and organisations voiced considerable support for the policy recommendations made in the NPHT report [14] as well as concern about the involvement of drinks industry groups in setting government policy related to alcohol, when the industry had previously opposed introduction of other potentially important policies [46]. However, the government rejected the volumetric tax proposal, citing a wine glut and possible industry restructuring [44]. Likewise, the government opted to only note recommendations regarding setting a minimum price for alcohol and restricting alcohol advertising through legislation, without committing to introduce the proposals [44].

Given these recent government responses, alcohol control policy advocates face challenges in arguing their case for reforms in these two core areas of policy, which are found to be highly contested public discussions [47,48]. This paper examines the views of key alcohol experts and advocates in Australia regarding priorities for the future of alcohol control in two policy areas: restrictions on alcohol advertising and regulating price via taxation and minimum pricing. We hypothesised that there would be strong consensus among experts over alcohol taxation, but that less coherence would be evident over alcohol advertising restrictions.

## Method

Potential informants were identified from members of the alcohol working group of the National Preventative Health Taskforce; policy advocates or researchers who repeatedly appeared in the Australian Health News Research Collaboration’s database [49] commenting on alcohol stories; and researchers who had recently published on alcohol policy. In addition, we identified policy specialists from websites for the Australian Drug Foundation [50], the National Drug Research Institute [51], the McCusker Centre for Action on Alcohol and Youth [52], the National Drug and Alcohol Research Centre [53], and the Foundation for Alcohol Research & Education [54]. Further recommendations were taken from participants as they were recruited.

### Data collection

We used semi-structured interviews conducted in person or via telephone using Skype when this was not possible. Interviews took between 45–60 minutes and audio was recorded and transcribed. Interviews involved open-ended questions about Australian alcohol policy priorities and detailed questions about two policy areas: taxation and restrictions on alcohol advertising.

The study protocol was approved by the University’s Human Research Ethics Committee (protocol #12034). All informants were given Participant Information Sheets, an opportunity to ask questions and signed Informed Consent forms prior to interview.

### Data analysis

Transcripts were analysed using NVIVO [55] to identify major themes related to informants’ views on policy priorities for alcohol in Australia. We approached the transcripts using concept-driven coding, where we created a coding scheme which followed topics outlined by the interview schedule, derived from best practice in prevention and also data-driven coding [56] where we allowed topics to emerge from reading the data, and these were added to the coding framework. Codes were trialled on three transcripts then used across all data. Our focus in this paper is primarily on points of agreement and disagreement between informants on tax and pricing policies and restrictions on alcohol advertising.

## Results

Twenty six subjects were invited to be interviewed and 21 (81%) participated. Respondents were university research academics (n=14) or employed by non-governmental and community-based organisations (n=7).

### General policy priorities

Participants were keen to emphasise the limited impact of pursuing any one area of policy change but of the need to pursue a range of options. *“You can’t just play with one piece of this puzzle; you have to play with all the pieces together if you’re really serious about changing culture” (E13).* The impact of any one policy was likely to be small and justified the need for a mix. *“They’re all going to have effects that are… not huge effects. So there’s no single one of them that will solve the problem…” (E11)*. Within that range of options, all agreed there were two to three policy areas which were critical to addressing problematic consumption of alcohol. “*The top three we push from a policy point of view are price, availability and marketing” (E13).* This sentiment was echoed by nearly all informants regarding price and availability, with some differences regarding the need to regulate alcohol advertising.

When trying to decide priorities for action, informants were influenced by different factors. Some emphasised the real-world constraints on policy making and the need to respond to changes in the policy environment that affected the likelihood of a specific policy being implemented. *“I haven’t bothered much to prioritise the strategies because policy making doesn’t happen in that way. You’ve got to be opportunistic. If something comes up…you’ve really got to jump on board and push” (E2).* An example of setting priorities based on the current political climate was raised by one informant: *“there is a tax forum… That should be our sector’s priority. We should be focussing on what the government is actually focusing on and not running off to lobby for changes to advertising regime” (E17).* In contrast, other informants might arrive at the same policy priority, but for different reasons. For example, evidence of effectiveness was often invoked as an imperative for action: *“There’s good evidence that pricing taxation is a very clear, logical way to go” (E18)*. Equally, lack of evidence of effectiveness was sometimes invoked as a caution, as in the case of advertising restrictions: *“I think we harm our good standing in the community if we start advocating things which we are advocating on the basis of instincts rather than evidence” (E9).* Another expert, speaking about the role of evidence in setting policy priorities said *“I want to be in a position where I’m telling the truth, even if it’s inconvenient.” (E11)* and emphasised that evidence should speak for itself, not be edited to fit within a policy argument.

### Policies to regulate the price of alcohol

All informants agreed that policies regulating the price of alcohol were of great importance and clearly supported by the international evidence base: “*it's been shown by the research around the world that it's the cheapest and easiest way to effect a population level consumption. It's a blunt instrument, it has to be conceded, but if you're ranking things on a one, two, three star it gets three stars” (E17).* Though price policies were viewed as having the most impact, informants still stressed the need for a range of policies: “*Taxation is the most cost effective but taxation doesn't work in isolation” (E3).* They also cautioned that while there was good evidence to introduce pricing policies, they did not always work exactly as predicted: *“So they're a tool. They're one of the more powerful tools but… the world is more complicated than simply saying you pull this lever and that happens” (E11).* Despite these cautions, informants argued that policies aimed at alcohol’s price were necessitated by (i) the harm created by cheap alcohol: “*I mean all those in the hospital, every week we've got people coming in drinking a couple of casks of wine a day. It's so cheap…” (E18)* and (ii) irregularities in the current taxation system, such as the wine equalisation tax [57], *“If we can fix that up then we've gone a long way to addressing those inequities of the system” (E3)*.

#### Volumetric taxation

Given these clear imperatives, informants regularly referred to two approaches to alcohol’s price that were most important for advocacy: *“…we need to get the volumetric and get it tiered so that there’s higher premium on higher alcohol products. Secondly, bring in a minimum price so we stop these two buck chucks being sold” (E13).*

Volumetric taxation was uniformly seen as a solution: *“it's consistency in taxation. So if people prefer to consume alcoholic beverages that have a high content they'll pay a higher price for it. If they prefer to drink low alcohol beverages it'll be cheaper. The industry will be guided by people's preferences” (E3).* It was also seen as a way to stop the drinks industry from exploiting loopholes that existed in the current taxation system: *“The industry's very clever…They diversify their products to suit and they'll target beverages that have a lower tax base to increase their profits” (E3).*

Some raised the idea that while a volumetric tax was essential, there should also be an option to apply higher taxes on particular drinks associated with greater harm in specific drinking contexts: *“…some sort of flexibility for a sort of harm tax as well. What I mean by that is to quickly identify any products that are particularly risky. For example if we’re finding that ready to drink alcohol is very sweet and attractive to young women… it actually is appropriate to… [apply] for want of a better term, a harm levy or a health dividend....” (E5).* Informants who raised this possibility did acknowledge some difficulties associated with the concept: *“…Is the trouble per litre different for different beverages? … It's really up in the air at this point. You can point to a couple of things around the edge where spirits really matters. One of them is obviously dying of an overdose. You really have to try hard to die of an overdose of beer” (E11).* Some highlighted the lack of evidence about specific drinks and the inability of existing data collection to answer such questions: *“So without that evidence it's very difficult to ascertain a relationship between what you drink and the particular harm associated with that” (E3).*

Nevertheless, one pointed to an agreement made between a collaboration of advocates that sought to reconcile taxes applied based on percentage alcohol with concerns this could make some spirits cheaper and increase harm: *“we agreed on a position which was sort of bands with higher tax per unit for a stronger beverage in about four or five bands” (E11).* This meant the tax applied would not be flatly tied to percentage alcohol by volume, but would be tiered in a number of tax bands that were progressively higher [31].

#### Minimum pricing

Some thought that the current political environment was unfavourable for volumetric taxation given that the Government was already under fire for introducing other “big taxes” in sectors other than health: *“… it would be portrayed as another great big new tax. I'm sure the Government's not going to touch it for that reason” (E16).* Given this concern, many proffered the idea of a setting a minimum ‘floor’ price. They reasoned that if a volumetric tax was politically untenable, then a minimum price would at least remove extraordinarily cheap alcohol from the market: *“… a minimum price as well to disallow grotesque promotions of cheap alcohol. So, you know, you buy a bottle of wine for $1.99 cheaper than you can buy a bottle of water” (E5)* and these informants saw minimum price as *“an alternative way of accomplishing much the same thing from a public health point of view” (E16).*

### Policies to regulate alcohol advertising

#### The role of evidence

All participants agreed that the nature and extent of alcohol advertising represented a clear challenge for policy reform: *“I think advertising matters. It matters in this long, public debate – an ongoing public debate about what’s the place of alcohol in the culture” (E11).*

However, beyond this basic level of agreement, there was some discordance over where to start and how the case for controls should be argued. In particular, there appeared to be disagreement regarding the evidence base for alcohol advertising’s impact on consumption. Some argued that *“Fundamentally the aggregate level data … it’s very difficult to show any effect of a change in advertising regulations on consumption…” (E11)* while others had a different view *“I reckon it’s pretty clear-cut because we don’t want kids to be exposed to that stuff and the research is clear that it increases consumption” (E13).* Some acknowledged that to date the evidence base had not been clear cut, but was now becoming clearer, especially in relation to young people: *“I mean, there's increasing evidence in the literature about the impact of advertising on young people. Branding, early uptake, familiarisation, you know, the use of Bundy Bears and kid friendly fuzzy looking creatures that kids warm to, all of this is an issue” (E6).* Some emphasised that evidence was difficult to collect in environments where branding and promotional activities were so pervasive “*the evidence for it as an etiological factor is not as strong as it is for the physical and economic availability just because of the nature of the intervention, the nature of the exposure. It's pervasive; it's hard to determine the effects of an increment in exposure to advertising, promotion and sponsorship” (E2).* Some felt the claim that there was no evidence was naive because comprehensive bans had *“never been tried” (E4).* Some suggested other strategies to address difficulties with the evidence base: *“These guys are selling a product which is a drug, which has an impact. I think the onus of proof should actually be more on them” (E5)* and asserted that the call to produce more evidence was disingenuous because “*advertisers do these things because they work” (E17)*.

#### Risk groups

Experts’ mostly concur that children and young people were the risk groups most susceptible to alcohol advertisements. There was consensus that policy advocacy on advertising controls should refer to those underage. Even where difficulties in reducing exposure were acknowledged, sentiment was strong: *“I think we need to ban any advertising to which kids are exposed and you look first and foremost at the high exposure areas for kids. So you're talking television, you're talking radio…You're talking very difficult … but you're talking internet” (E4).*

Some extended the focus to advertising targeting older high risk groups *“I think we know the young males who are high risk group, and the young males are the ones who are most likely to watch sport and be influenced by sporting heroes” (E18).*

### What kind of restrictions?

Respondents discussed a range of options to consider. There was no clear consensus on what the first steps should be, or the form that advertising regulations should take if policy reform opportunities arose. Some favoured legislation of existing ABAC guidelines, with an independent body to oversee restrictions: *“…the objective here is to get the industry's code, which is by and large not a bad code - it's just that nobody observes it - to get that converted into a statutory form, legislated and to get rid of some of the exemptions that the industry provides for itself” (E17).* Others thought that advertising content was almost irrelevant and it was more important to regulate where and how often alcohol advertising could appear. *“I think there's potentially a persuasive public case to reduce advertising and to move it from certain locations or certain associations” (E19).* Still others thought this missed the point: *“Look I think it's a good idea. But while we've got sponsorship of sporting heroes, that totally unravels it, because you've associated it with fit, muscly young guys. So it's undercover doing exactly the same” (E18).*

Again, these differences were related to whether they approached problems pragmatically, with an eye to the current political climate, or whether they centred on the evidence base as their guide: *“My reading of the evidence is that restrictions on what is in the ad are almost useless. That it’s the amount of advertising that matters, if anything matters” (E11).* Other respondents were influenced by personal circumstances, such as their experiences with children: “*As a parent I struggle with the fact that I don't have the freedom to sit down and watch cricket… and have my kids watch it and for them - I don't have the choice as to whether they're going to be exposed to alcohol advertising or not. That really bothers me” (E2).*

These differences were common and an awareness of them within the alcohol control field was acknowledged by most respondents throughout their interviews. In acknowledging these differences, they also recognised many difficulties associated with introducing regulations, particularly with regard to sporting promotional activities. Many agreed that exposure to alcohol advertising through professional sport was a problem to be addressed and concern was particularly concentrated on television advertisements: *“I think the first transgression is the code essentially says, no advertising of alcohol products before 8.30 pm, except if it's sport. Well on the weekends it’s wall to wall sport” (E17).*

Despite agreement among experts that “something should be done” many identified challenges to implementing any meaningful change in this arena, especially if all sporting groups in Australia were considered. Some were concerned about the practicalities of how to phase out sponsorship “…*probably it's necessary to phase it out over a number of years and give the sports the chance to find other sources of revenue” (E2).* Some were more concerned about defining exactly what ‘sponsorship’ meant, particularly for non-professional teams. For example, “*you can certainly fuss around with what they’re allowed to do and not allowed to do. Are they allowed to have a cap that says VB on it?” (E11).* Such concerns gave way to the effect this might have on sporting teams in smaller towns: “*… in some places that's all the support that the local sports club gets. Sometimes there's no other sponsor big enough in the town” (E16).* When prompted to talk about the potential for government buyouts of existing sponsorships, other problems were highlighted: *“I think the problem we’ve got is we don’t understand how much money is required…it’s the value to the TV stations and the value then to the media rights, so you’re not talking small quantities of money” (E13).* Ethical issues were also raised: “*if you're doing that at the club level, you're actually rewarding the sports clubs that have taken on an alcohol sponsorship and you're giving nothing to the clubs that have battled on without one” (E13).*

### The alcoholic beverages advertising code

All respondents were unanimous in their assessment that the current system of voluntary, self-regulated, industry codes of practices were not functioning as they should. The ABAC guidelines and their implementation were variously called a *“joke” (E2), “rubbish”* and a *“sham” (E6),* a *“stunt for industry”* (E13), *“protection devices for the industries… no teeth at all” (E11)* and *“so weak that you can’t do anything with them…” (E4).* This sentiment was expressed often, alongside dismay that the government did not move to address the issues: *“They’ve had two government inquiries into that, both inquiries have said it doesn’t work and both governments of different persuasions have said we’re going to keep it going. What the hell is going on?” (E13).* Others pointed out that the current system relies on members of the public making a complaint, which means exposure to alcohol promotion is not reduced until adjudication has been made.

### New media

Some mentioned forms of new media that current guidelines did not cover: *“you have to pay attention to the whole range, including what they call below the line stuff, the stuff that is viral advertising on the web and so forth” (E11).* They emphasised the role of technology “*it’s now not just about a few adverts on television. It’s about the viral stuff, the iPhone applications, the stuff on the internet that is just passing by a lot of people” (E5)* and they positioned social media networks as places where underage people were exposed to unrestrained and engaging alcohol advertising: *“the internet's totally unregulated, and there are very effective advertisements on the internet. All the 16 year olds are all logging onto Facebook pages for the Goon Bags, and The Big Beer Ad which was so good that I watched it several times” (E18).*

## Discussion

Our study considered accord among Australian alcohol policy researchers and advocates about two core alcohol control policy options. Our findings offer insights from experts that may assist future advocates to join the public conversation and for all advocates to present a united front, arguing the case for evidence based pragmatic solutions for alcohol related problems.

All informants readily agreed that price is a key focal area for reducing alcohol problems. However, the consensus among those interviewed about the regulation of alcohol advertising and promotion was comparatively “general”: when prompted to discuss explicit restrictions on advertising, differences emerged. While there was broad consensus that “something needs to be done” about controlling alcohol promotions, there was little consensus on exactly what should occur and with what priority. This is echoed in public discussions, where news-coverage of alcohol advertising controls shows similar divergence of opinion concerning what kind of advertising is the focus and what form regulation should take [47]. While the National Alliance for Action on Alcohol (NAAA) now has a clear position statement on advertising reform [58], the organisation was in its early stages at the time of interview and these suggestions had yet to result in a clear ‘cut through’ message that advocates repeatedly endorse in public statements or the private discussions reported here. The field would thus benefit from wider sectoral agreement on a policy platform on advertising controls, specifying and arguing for an explicit set of reforms, with the NAAA being well placed to support such activity.

The high degree of unity within the alcohol control field regarding the importance of a volumetric tax is notable. While informants expressed dismay that the current political climate was unsupportive of this measure, the consensus causes consistent messages to be voiced on this issue. That said, there was some lack of agreement over whether the tax should follow a flat rate based purely on volume of alcohol, or whether there should be allowances made for higher taxing in higher bands tied to volume. Thus, advocacy efforts focussed on existing policy platforms articulated by the NPHT and the NAAA regarding the likely form a banded-volumetric tax would take may provide important reference points to “sign on” all organisations which support reform [21]. While the introduction of a volumetric tax is not imminent, the consensus about reform on this issue among researchers and advocates is unlikely to see it disappear as a repeated core demand when alcohol advocates repeatedly provide public news commentary on “solutions” to alcohol problems in the community.

The rate of progress of policy reform in other fields provides encouragement for ongoing long-term advocacy despite the political conditions. For example, plain tobacco packaging legislation has been recently introduced after first being proposed in 1986 [59]. Clearly, advocacy for volumetric taxation will require a long-term commitment to continuing advocacy, while producing the evidence necessary to arguing the case.

Our findings also suggest a clear opportunity for a united vision regarding minimum or floor pricing of alcohol to emerge in the public debate. Some informants pointed out that public health improvements to be gained from such a policy are clear and evidence based [60,61]. Given the current assessment that a volumetric tax is unlikely, minimum pricing proposals potentially represent an alternative strategy for those advocates who support pragmatic solutions which may have a greater chance of being adopted in a given political climate. Minimum pricing for alcohol has been trialled in the Northern Territory and is the focus of a current issues paper for the Australian National Preventive Health Agency [62]. However, minimum pricing is contested by sectors of the drinks industry, who contend that a minimum price would adversely affect moderate drinkers and low-income households while having limited impact on heavy drinkers [28]. There are also questions about whether minimum price policy would be considered ‘anti-competitive’ and breach existing trade agreements. This was found not to be the case in the UK and is seen as viable in Australia [63]. Clarification of these points represents a substantial opportunity for united advocacy in the future.

At present, the government has rejected outright proposals to introduce a volumetric tax yet has opted for ‘continued monitoring’ of alcohol advertising. While this is far from ideal for those urging the introduction of legislated controls on advertising, the possibility of such measures being introduced in a subsequent, more favourable political climate remains. Focusing on greater specification about the message concerning alcohol advertising may be a key strategic move for Australian advocates.

Previously, this issue received scant and poorly focussed news coverage [47]. The NPHT report recommended that an initial focus on restricting alcohol advertising should attend specifically to underage exposure and promotions associated with sport. Our interviews did not show consensus on the same focus, with differences of opinion about priority risk groups, whether it was the timing, frequency or content of advertising that should be addressed nor on how to approach the issues of alcohol sponsorship in sports beyond a very general concern that it was a problem. Only one respondent reported an organisational focus on ending sports sponsorships in line with the NPHT recommendations. There is currently no national policy platform that appears to be supported publicly by all leading figures in the field, despite national recommendations and a clear policy position adopted by the NAAA. Securing the understanding and support of such agencies for these national policies would ensure a more clearly articulated vision about the specific controls on advertising that are needed.

Informants were clear that the implementation of the ABAC guidelines were not effective in reducing underage exposure to advertising. Capitalising on this consensus will be a sensible point of reference in stimulating further, more focussed and precise policy discussion. Indeed, the Alcohol Advertising Review Board [64] is also adjudicating complaints about alcohol advertising, providing a point of comparison to a drinks industry administered mechanism where complaints are assessed and rejected at a high rate [38,42]. This seems likely to further highlight the problems with the current self-regulatory system. Although this alternative adjudication system has no powers to order changes in advertising, nor does the ABAC system, and it remains a useful tool to highlight the major shortcomings of the present industry self-regulation.

A key area of concern for regulation of alcohol advertising is the internet and its social media networks. While the NAAA mentions the internet in its policy priorities, the current NPHT recommendations do not address online alcohol advertising. This neglect was raised by few informants in our study. There are indications that pages with branded material on social networks like Facebook are becoming more common and recently were found by the ASB to be a form of marketing that falls under the ABAC guidelines [65]. This is an important ruling for alcohol control, and should stimulate alcohol control agencies to develop policy positions on internet promotions. There are several examples of explicit policy priorities that advocates and experts in the field of alcohol control can consider [14,58]. However, the current differences reported here indicate that not all Australian alcohol control agencies are united on what these should be and ensuring that all members of the sector promote the same vision. Some have argued that an internationally agreed framework convention on alcohol control is of high importance [66] in achieving clearly articulated and agreed upon policy reform steps.

Some limitations to interpreting the results of this study include the fact that not all experts identified could participate and we note there may be candidates for inclusion who were not identified by our sampling approach. While we made efforts to identify experts in public roles regarding alcohol advocacy, it is possible that further input from other experts could advance the current discussion. Likewise, other research foci were covered in the interviews that could not all be included here and restricting the focus of this paper to pricing and promotions policies may have limited the scope of these findings. We also note that after the research was conducted, new policy alliances were created that are working towards common policy goals, for example, the NSW/ACT Alcohol Policy Alliance (NAAPA) [67]. Likewise, the aforementioned National Alliance for Action and Alcohol [21] and the Alcohol Advertising Review Board [64], though formed, were still in the early stages of refining policy positions when the research was conducted. It is likely that such organisations are already assisting to move the sector forward on unified approaches to alcohol policy solutions.

Finally, our findings point to a need for further research to answers questions about the evidence base, which could be used by advocates in future policy advocacy. Namely, clearer answers are required about whether harm-taxes for specific drinks are warranted, what impact advertising restrictions would have, whether any restrictions short of a total ban would have the desired impact, and the kind of sporting sponsorship that should be tackled first. These would help to clarify and unite positions taken by experts across the whole field.

## Conclusions

There is a high degree of unity among alcohol experts regarding the policy priorities for alcohol control in Australia, with recognition of substantial challenges to be faced in implementing reform. While experts agree in principle with needed reforms to alcohol’s price and promotion, our findings suggest there is still room for greater agreement on the details and specific forms these policies would take in the future, particularly with regard to alcohol advertising. With these finer details resolved, potential advocates throughout the sector could confidently add their voices to experts’ voices in existing public discussions.

## Competing interests

The authors declare that they have no competing interests.

## Authors’ contributions

ASF conceived of the study, designed the study protocol, collected all data, performed the analysis, and drafted and revised the manuscript. SC supervised the study’s conception, design and data collection, and helped to draft and revise the manuscript. All authors read and approved the final manuscript.

## Pre-publication history

The pre-publication history for this paper can be accessed here:

http://www.biomedcentral.com/1471-2458/13/610/prepub
